# Shubnikov - de Haas oscillations, weak antilocalization effect and large linear magnetoresistance in the putative topological superconductor LuPdBi

**DOI:** 10.1038/srep09158

**Published:** 2015-03-17

**Authors:** Orest Pavlosiuk, Dariusz Kaczorowski, Piotr Wiśniewski

**Affiliations:** 1 Institute for Low Temperatures and Structure Research, Polish Academy of Sciences, P. O. Box 1410, 50-950 Wrocław, Poland

## Abstract

We present electronic transport and magnetic properties of single crystals of semimetallic half-Heusler phase LuPdBi, having theoretically predicted band inversion requisite for nontrivial topological properties. The compound exhibits superconductivity below a critical temperature *T*_c_ = 1.8 K, with a zero-temperature upper critical field *B*_c2_ ≈ 2.3 T. Although superconducting state is clearly reflected in the electrical resistivity and magnetic susceptibility data, no corresponding anomaly can be seen in the specific heat. Temperature dependence of the electrical resistivity suggests existence of two parallel conduction channels: metallic and semiconducting, with the latter making negligible contribution at low temperatures. The magnetoresistance is huge and clearly shows a weak antilocalization effect in small magnetic fields. Above about 1.5 T, the magnetoresistance becomes linear and does not saturate in fields up to 9 T. The linear magnetoresistance is observed up to room temperature. Below 10 K, it is accompanied by Shubnikov-de Haas oscillations. Their analysis reveals charge carriers with effective mass of 0.06 *m_e_* and a Berry phase very close to *π*, expected for Dirac-fermion surface states, thus corroborating topological nature of the material.

Large family of half-Heusler compounds that crystallize in a noncentrosymmetric cubic MgAgAs-type structure attracts much attention due to their remarkable magnetic and electrical transport properties^1^,^2^. Recent *ab initio* electronic structure calculations have predicted that several dozen of half-Heusler compounds, due to a strong spin-orbit coupling, have inverted bands requisite for topological properties^3^,^4^,^5^. Topological insulators (TI) constitute a new class of materials, which are insulating in the bulk, but at the same time their surface states are protected from backscattering by Z_2_ topology^6^,^7^,^8^. These surface states are theoretically described as massless Dirac fermions with linear dispersion and lifted spin-degeneracy. Such properties open new prospects for spintronic applications. In contrast to two-dimensional (2D) TIs, like HgTe, some of half-Heusler phases possess not only band inversion at an odd number of time-reversal-invariant momenta, but also may have a bulk band-gap opening under uniaxial pressure, which lowers the crystal symmetry^9^. In result, the rare-earth (*RE*) based half-Heusler compounds (*RE*PdBi, *RE*PtBi, *RE*PdSb, *RE*PtSb) may possibly form the biggest group of the three-dimensional (3D) TIs.

After the discovery of the 3D TI systems, a quest for superconductivity in materials with the non-trivial topology of electron bands started due to their potential applications in topological quantum computing^10^,^11^,^12^. Topological superconductor (TS) is a material characterized by protected Majorana surface states and bulk consisting of mixed-parity Cooper-pair states^13^,^14^. The first theoretically forecasted and experimentally discovered TS was Bi_2_Se_3_ doped with Cu atoms^15^,^16^. Since it remains problematic to clarify the nature of the surface Majorana fermions in the inhomogeneous crystals of Cu*_x_*Bi_2_Se_3_ (Ref. 17), new TS materials are sought extensively among low-carrier-density semiconductors, whose Fermi surfaces are centered around time-reversal-invariant momenta^18^. In this context, the superconductivity recently found in the bismuthides *RE*PtBi: LaPtBi (*T*_c_ = 0.9 K)^19^, LuPtBi (*T*_c_ = 1.0 K)^20^, and YPtBi (*T*_c_ = 0.77 K)^21^,^22^,^23^ appears most attracting. Besides, the content of magnetic rare-earth may bring about other properties, such as long-range antiferromagnetic ordering or heavy fermion behavior^24^.

Potentially, the rare-earth palladium-bearing bismuthides *RE*PdBi are equally interesting from the point of view of topological and superconducting properties. Magnetic and transport behaviors of these compounds have been first investigated on polycrystalline samples^25^,^26^,^27^,^28^. Most of them have been characterized as local-moment antiferromagnets (with Néel temperatures *T*_N_ = 2–13 K) with electrical properties characteristic of semimetals or narrow-band semiconductors. Recently, diamagnetic YPdBi has been reported to exhibit large linear magnetoresistance (LMR) and Shubnikov-de Haas (SdH) quantum oscillations at low temperatures, and its nontrivial topological nature has been evaluated^29^. In turn, CePdBi has shown some features of incipient superconductivity (*T*_c_ = 1.3 K) emerging in the antiferromagnetic state (*T*_N_ = 2 K), and a hypothesis on topological character of the compound has also been formulated^30^. Similarly, ErPdBi has been reported to show the coexistence of antiferromagnetism (*T*_N_ = 1.06 K) and superconductivity (*T*_c_ = 1.22 K), and hence acclaimed as a new platform for the research of the reciprocity of magnetic order, superconductivity, and nontrivial topological states^31^. However, in our own study on single-crystalline ErPdBi, we could not confirm those findings because no clear-cut evidence for any intrinsic superconducting state in this material has been observed^32^. Most recently, superconductivity (*T*_c_ = 1.7 K) has been reported for nonmagnetic LuPdBi, accompanied by a 2D weak antilocalization (WAL) effect^33^. Since the latter feature is considered as a fingerprint of topological surface states, the compound was suggested to be a topological superconductor with Majorana edge states^33^. In this work, we present the results of our comprehensive magnetic susceptibility, specific heat and electrical resistivity measurements performed on high-quality single crystals of LuPdBi. The obtained data are here critically compared with those reported in Ref. 33.

## Results

### Electrical resistivity and Hall effect

**Figure 1:**
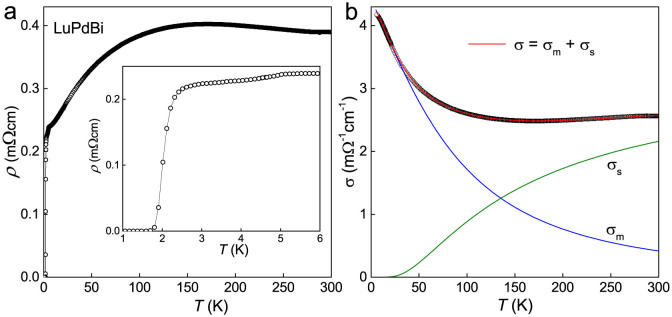
Electrical transport properties of single-crystalline LuPdBi. (a) Temperature dependence of the electrical resistivity up to 300 K. Inset: low-temperature resistivity data revealing a superconducting transition near 2 K. (b) Temperature dependence of the electrical conductivity in the normal state fitted by the function *σ*(*T*) = *σ*_m_(*T*) + *σ*_s_(*T*) (red line) described in the text. The metallic and semiconducting contributions are represented by blue and green curves, respectively.

The temperature dependence of the electrical resistivity, *ρ*, of the LuPdBi single crystal is shown in Figure 1a. From 300 K down to ≈170 K, the resistivity displays a semiconducting-like character (d*ρ*(*T*)/d*T* < 0), then at lower temperatures *ρ*(*T*) becomes typical for metals. Such a behavior of the resistivity is common for the *RE*PdBi compounds^26^,^27^,^28^,^31^,^32^. As demonstrated in Figure 1b, the overall behavior of the electrical resistivity in LuPdBi can be well described over an extended range of temperature considering two independent channels for charge transport: semiconducting- and metallic-like. The former contribution is written as *ρ*_s_(*T*) = *a* exp(−*E_g_*/*k*_B_*T*), where *E_g_* is an energy gap between valence and conduction bands, while the latter one is expressed as *ρ*_m_(*T*) = *ρ*_0_ + *bT*^2^ + *cT*, where *ρ*_0_ is the residual resistivity due to scattering on structural defects, the second term represents electron-electron scattering processes, while the third one accounts for scattering on phonons. The total conductance, *σ*(*T*) ≡ 1/*ρ*(*T*) is the sum *σ*(*T*) = *σ*_s_(*T*) + *σ*_m_(*T*), where *σ*_s_(*T*) ≡ 1/*ρ*_s_(*T*) and *σ*_m_(*T*) ≡ 1/*ρ*_m_(*T*). Fitting these expressions to the experimental data of LuPdBi in the wide temperature interval from 5 K to 300 K, yielded the parameters: *a* = 296 mΩcm, *E_g_* = 11.5 meV, *ρ*_0_ = 226 mΩcm, *b* = 0.018 mΩcm/K^2^, and *c* = 1.72 mΩcm/K. The so-derived energy gap *E_g_* is very close to 15.2 meV reported for LuPdBi in Ref. 25, and very similar to the values reported for other *RE*PdBi phases^26^,^27^,^28^,^31^,^32^.

It is worth noting that the temperature variation of the resistivity of LuPdBi single crystal obtained in the present work somewhat differs from that reported before by Xu et al.^33^, who observed a monotonous increase of the resistivity with decreasing temperature from 300 to 2 K, and attempted to analyze their *ρ*(*T*) data above 90 K in terms of 3D variable-range hoping model. However, also in that case, the transport at low-temperatures was attributed to the metallic channel (though no quantitative analysis was made).

**Figure 2:**
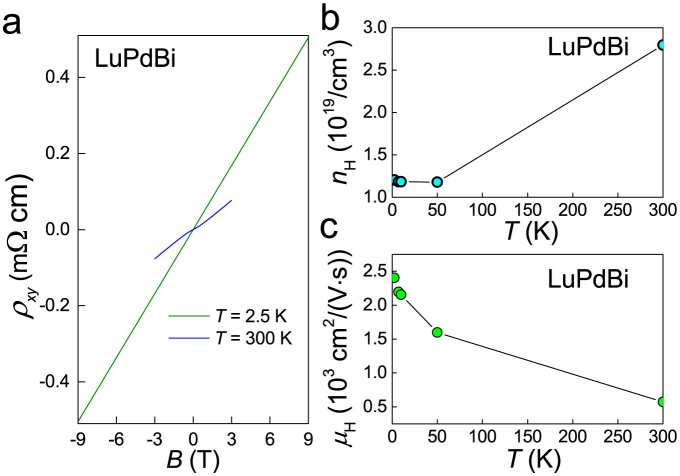
Hall effect in LuPdBi. (a) Magnetic field dependence of the Hall resistivity measured at 2.5 and 300 K. (b) Temperature variation of the Hall carrier concentration. (c) Temperature variation of the Hall carrier mobility.

As displayed in Figure 2a, the Hall resistivity of LuPdBi measured at 2.5 K is a linear function of the applied magnetic field up to 9 T. The Hall coefficient evaluated from this data was *R*_H_ = 0.0565 mΩcm/T. Almost identical magnitude of *R*_H_ was derived from *ρ*_xy_(*B*) collected at *T* = 4, 7, 10 and 50 K (not shown). Assuming a single parabolic band, one may estimate the Hall carrier concentration, *n*_H_, to be at low temperatures 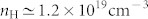. Above 50 K, *n*_H_ was found to increase with increasing temperature and to attain at 300 K a value of 2.8 × 10^19^ cm^−3^ (see Figure 2b). In turn, as demonstrated in Figure 2c, the so-obtained Hall carrier mobility, *μ*_H_, gradually decreases with increasing temperature, from the value 2404 cm^2^V^−1^s^−1^ at *T* = 2.5 K to 573 cm^2^V^−1^s^−1^ at 300 K.

The inset to Figure 1a displays the resistivity of single-crystalline LuPdBi at the lowest temperature studied. In agreement with Ref. 33, the compound undergoes a transition to superconducting state. The resistivity starts to drop near 2.2 K and becomes zero below 1.8 K. Using a criterion of 90% drop in the resistivity value from the point where the *ρ*(*T*) curve starts bending down, *T*_c_ = 1.9 K was determined. This value is slightly higher than *T*_c_ = 1.7 K reported before for LuPdBi (Ref. 33), and remains the highest *T*_c_ found so far for the *RET*Bi superconductors. As can be inferred from the inset to Figure 1a, besides the superconducting phase transition, we observed a small resistivity drop below 5 K. The existence of a fairly similar anomaly in *ρ*(*T*) near this temperature has previously been reported for a few other half-Heusler bismuthides and antimonides^26^,^34^, and tentatively attributed to superconductivity of thin films of metallic Bi(Sb) located at grain boundaries, yet the actual nature of this feature remains unclear.

**Figure 3:**
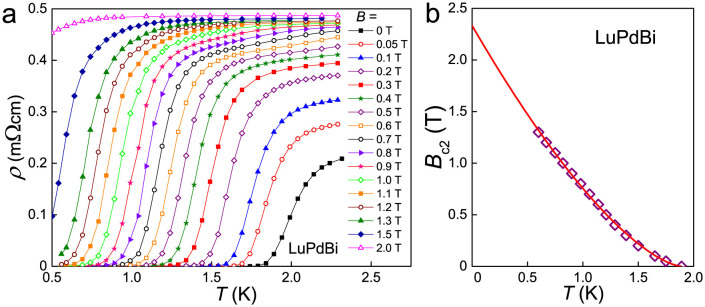
Superconductivity in LuPdBi. (a) Low-temperature dependencies of the electrical resistivity measured in different external magnetic fields. (b) Upper critical field as a function of temperature. Solid line represents the fit of the function 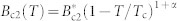 described in the text.

The temperature dependence of the electrical resistivity of LuPdBi measured in external magnetic fields up to 2 T is shown in Figure 3a. The width of superconducting transition increases gradually from 0.4 K at zero field to 0.7 K at 1 T. The critical temperature defined by the drop of the resistivity to 90% of its normal-state value varies with magnetic field in a manner presented in Figure 3b. The so-derived *B*_c2_(*T*) dependence has a positive curvature, characteristic of two-band clean-limit type-II superconductors, like YNi_2_B_2_C, LuNi_2_B_2_C (Ref. 35) or MgB_2_ (Ref. 36). Though our sample exhibits the residual resistivity ratio *ρ*(300 K)/*ρ*(4 K) of only 1.8, the observation of SdH oscillations described below, indicates its very good quality, which allows us to consider the observed superconductivity to have a clean limit character. Indeed, as displayed in Figure 3b, the experimental *B*_c2_(*T*) data can be well approximated in the whole temperature range by the expression 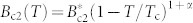, previously applied, e.g., to MgB_2_ (Ref. 36). The fitting parameter 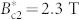 can be considered as the upper limit for the upper critical field *B*_c2_(0). The parameter *α* = 0.496(22) is significantly larger than 0.32 and 0.24 (0.25) reported for MgB_2_ and LuNi_2_B_2_C (YNi_2_B_2_C), respectively^35^,^36^. Using the relation 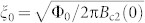, one obtains an estimate for the coherence length *ξ*_0_ = 17 nm. This value is about three times larger than those reported for MgB_2_ (Ref. 36), YNi_2_B_2_C and LuNi_2_B_2_C (Ref. 37). Nevertheless, it is still distinctly smaller than the mean free path *l* = 60 nm derived from the SdH data (see below) and this comparison corroborates our assumption that the superconductivity in LuPdBi occurs in the clean limit (*l*/*ξ*_0_ > 1). The Werthamer-Helfand-Hohenberg approximation^38^ provides an estimate for the orbitally limited upper critical field 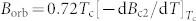 to be 0.5 T. The Pauli limited upper critical field *B*_P_ can be evaluated from the equation 
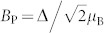
, where the BCS energy gap Δ = 1.76 *k*_B_*T*_c_. The estimate for the measured crystal of LuPdBi is *B*_P_ = 3.5 T. Thus, the Maki parameter *α*_M_ = *B*_orb_/*B*_P_ = 0.2 was obtained. One can conclude that the superconductivity in LuPdBi is Pauli limited. It is worthwhile recalling that the relationship *B*_orb_ < *B*_c2_ < *B*_P_ was established also for the related half-Heusler superconductors YPtBi and LuPtBi (Refs. 20 and 22).

### Thermodynamic properties

**Figure 4:**
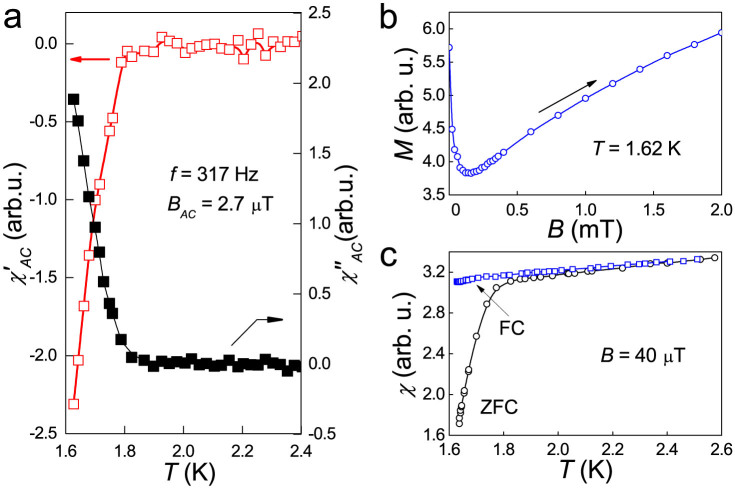
Magnetic properties of single-crystalline LuPdBi. (a) Temperature variations of the real and imaginary components of the AC magnetic susceptibility taken with a magnetic field of 2.7 *μ*T alternating with a frequency of 317 Hz. (b) Field dependence of the magnetization measured at *T* = 1.62 K upon cooling the specimen in zero magnetic field. (c) Low-temperature dependence of the DC magnetic susceptibility recorded upon cooling the specimen in zero magnetic field (ZFC) and in an applied field of 40 *μ*T (FC). All solid lines are guides to the eye.

Figure 4 displays the results of magnetic measurements performed on a collection of several single crystals of LuPdBi. Both components of the AC magnetic susceptibility show clear anomalies at 1.8 K typical for a superconducting transition. The corresponding feature is seen on the temperature dependence of the DC magnetic susceptibility measured in a weak magnetic field upon cooling the sample in zero field. Comparison with the data obtained in field-cooled regime reveals superconducting current screening effect. Furthermore, the magnetization *M* measured at the lowest temperature *T* = 1.62 K attainable in our experimental setup exhibits a field variation characteristic of type-II superconductors. All these features are in concert with the electrical resistivity data and manifest the emergence of the superconducting state in LuPdBi below *T*_c_ = 1.8(1) K. From the *M*(*B*) curve presented in Figure 4b one can roughly estimate the upper limit for the lower critical field *B_c_*_1_ to be of 0.05 mT. Then, the formula 
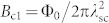
 yields the penetration depth *λ*_sc_ = 3629 nm.

**Figure 5:**
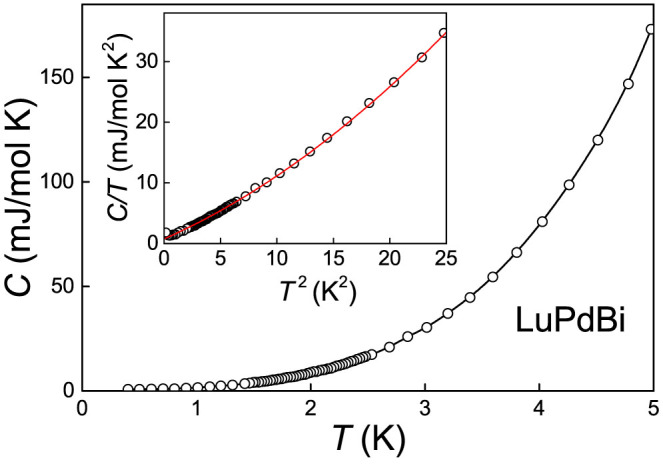
Heat capacity of LuPdBi. Low-temperature dependence of the specific heat. Inset: specific heat over temperature ratio as a function of temperature squared. Solid red line represents the fit to the function *C*/*T* = *γ* + *βT*^2^ + *δT*^4^ discussed in the text.

As can be inferred from Figure 5, the low-temperature dependence of the specific heat of LuPdBi is featureless. In particular, no obvious anomaly is seen at the superconducting phase transition. Remarkably, analogous situation occurs for other half-Heusler superconductors: LuPtBi (Ref. 39) and YPtBi (Ref. 40). Lack of any feature in *C*(*T*) at *T*_c_ in these systems may be due to the fact that superconductivity originates from topologically protected surface states, which occupy a very small volume in relation to the volume of entire sample. The only exception is the result reported by Xu et al.^33^ who observed for their single crystal of LuPdBi a prominent BCS-like specific heat jump at the onset of superconductivity. A critical comment on this data can be found in Supplementary Material.

As shown in the inset to Figure 5, below 5 K, the *C*/*T*(*T*) variation can be well described by the standard formula *C*/*T* = *γ* + *βT*^2^ + *δT*^4^ with the electronic specific heat coefficient *γ* = 0.75 mJ/mol K^2^ and the coefficients of the phonon contribution *β* = 0.8 mJ/mol K^4^ and *δ* = 0.02 mJ/mol K^6^. From the relation Θ_D_ = (12*nRπ*^4^/5*β*)^1/3^ (*R* is the gas constant and *n* is the number of atoms per formula unit) one derives the Debye temperature Θ_D_ = 194 K. The obtained values of *γ* and Θ_D_ are close to those reported in Ref. 25: *γ* = 0.096(327) mJ/mol K^2^ and Θ_D_ = 194.9 K but strongly disagree with the results derived for LuPdBi by Xu et al.: *γ* = 11.9 mJ/mol K^2^ and Θ_D_ = 295 K (Ref. 33). It is worth recalling that for other nonmagnetic *RET*Bi the values of *γ* are also very small: 0.1 for YPtBi (Ref. 41) and 0.06 mJ/mol K^2^ LuPtBi (Ref. 39).

### Magnetoresistance

**Figure 6:**
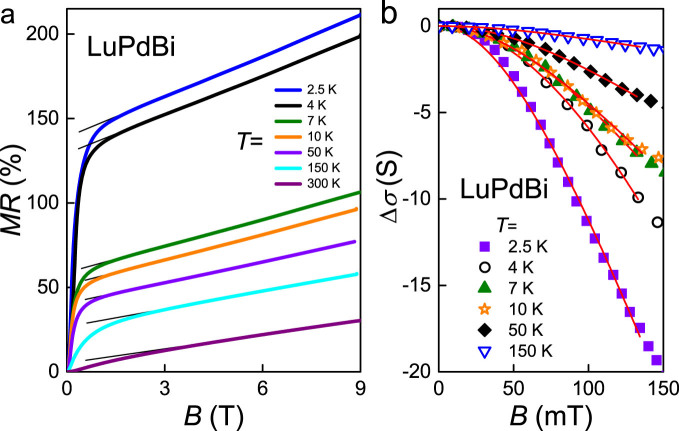
Transverse magnetotransport in single-crystalline LuPdBi. (a) Field dependence of the magnetoresistance measured at different temperatures. Solid lines emphasize the linear behavior. (b) Low-field variations of the conductance measured at different temperatures. Solid curves represent the functions given in Eq. 1, which account for the weak antilocalization effect.

Figure 6a shows the magnetoresistance data of LuPdBi, defined as MR(*B*) = [*ρ*(*B*) − *ρ*(0)]/*ρ*(0)], with *ρ*(*B*) and *ρ*(0) denoting the electrical resistivity measured in applied field and in zero field, respectively. Remarkably, at 2.5 K, MR is as large as 210% in the strongest field of 9 T achievable in our experimental setup. With increasing temperature, MR gradually diminishes, yet even at 300 K it reaches about 30% in *B* = 9 T, which is a very large value. A striking feature of MR(*B*) is its linear nonsaturating behavior. Above a certain magnetic field (ranging from 1.5 T at 2.5 K to 3 T at 300 K), MR becomes proportional to *B* with a slope systematically decreasing with increasing temperature, from about 8%/T at the lowest temperatures, down to 3%/T at 300 K.

In conventional theory of metals and semiconductors, MR should grow quadratically in weak magnetic fields and then saturate in strong fields^42^. Linear magnetoresistance (LMR) is a phenomenon reported for narrow-gap semiconductors^43^,^44^,^45^, multi-layered graphene^46^, topological insulators^47^,^48^,^49^ and Dirac semimetals^50^,^51^. Two main approaches were made to understand this behavior. In their classical theory, Parish and Littlewood attributed LMR to material inhomogeneities, which give rise to sturdy spatial fluctuations in the electrical conductivity^52^. In turn, Abrikosov developed the quantum magnetoresistance theory^53^, that predicts LMR in zero-gap band systems with linear energy dispersion being in the extreme quantum limit (EQL) in which all electrons are confined to the first Landau level. The Abrikosov theory was demonstrated to work correctly for graphene^46^ and topological insulator Bi_2_Se_3_ nanosheets^47^, with topological surface states possessing a linear energy dispersion over wide temperature interval.

For single-crystalline LuPdBi, the detailed X-ray and microprobe characterization revealed a very good sample quality, which was further confirmed by the observation of SdH oscillations (see below). This prompts us to dismiss the classical theory. On the other hand, the compound shows LMR behavior virtually identical to that reported for silver chalcogenides, interpreted within the quantum magnetoresistance theory^53^. Moreover, the SdH data indicated that the studied crystal was in an EQL regime at temperatures up to at least 10 K. All these findings suggest that the quantum approach is perfectly applicable, and the observed LMR effect is a hallmark of Dirac-like band structure in this putative topological semimetal.

In weak magnetic fields, MR of LuPdBi displays a very abrupt rise. For example, at *T* = 2.5 K, MR attains a huge value of 150% already in a field of 1.5 T. Similar situation, but with lower MR increase, occurs at higher temperatures, up to 150 K. Such sharp cusps on the MR isotherms are reminiscent of a weak antilocalization (WAL) effect, which relies on destructive interference of the electron wave functions between two closed paths with time-reversal symmetry. In topologically nontrivial materials, WAL is enhanced due to ability of topologically protected surface states to collect a *π* Berry phase due to the helical spin polarization. Thus, the WAL effect may be considered as a manifestation of surface-state transport in LuPdBi.

Taking into account the coexistence in our LuPdBi sample of two parallel conducting channels (see Figure 1b and the discussion above), in conjunction with the plausibly topological nature of the compound, one may assume that at temperatures below ≈25 K the conductivity was almost entirely determined by the metallic channel due to the sample surface. Consequently, the sheet resistance *R_s_*(*B*) of the conducting surface of the sample can be derived. Figure 6b displays the sheet magnetoconductance of LuPdBi, Δ*σ*(*B*) = (1/*R_s_*(*B*)) − (1/*R_s_*(0)), where *R_s_*(*B*) and *R_s_*(0) represent the data obtained in applied field and zero field, respectively. These experimental results can be analyzed in terms of the Hikami-Larkin-Nagaoka formula^54^
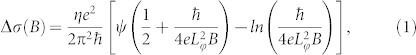
where *L_φ_* stands for a phase coherence length, *η* is a parameter depending on the strength of spin-orbit interaction and magnetic scattering, *ψ*(*x*) is digamma function, while the other symbols have their usual meanings. For *T* = 2.5 K, the fitted parameters are *L_φ_* = 49.6(2.2) nm and 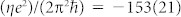 and are comparable to *L_φ_* ≈ 95 nm and 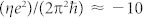 obtained for the same compound in Ref. 33. This finding strongly supports our assumption of the 2D topological nature of the metallic conducting channel in LuPdBi. For higher temperatures, where the contribution due to the bulk channel becomes noticeable, the fits of Eq. 1 to the experimental data are still of good quality, but the value of *η* decreases with increasing temperature. More detailed description of the dependence of WAL parameters on temperature is given in Supplementary Material. It is worth mentioning that a similar analysis of MR(*B*) of LuPdBi was presented in Ref. 33, however the fitted values of *η* were ≈10^5^ larger than that expected for a TI system (see also our comment in Supplementary Material).

### Shubnikov-de Haas oscillations

**Figure 7:**
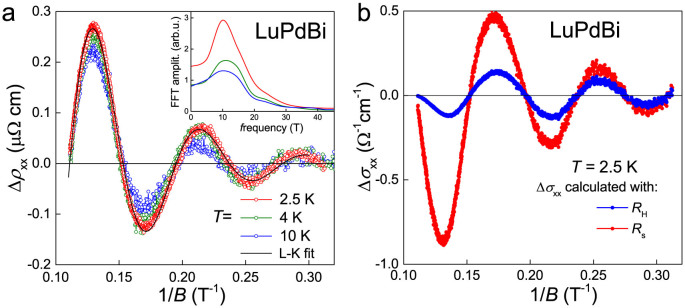
Shubnikov–de Haas oscillations in LuPdBi. (a) Oscillatory component of the electrical resistivity as a function of inverse magnetic field, measured at different temperatures. Black solid line represents a fit of 2.5 K data with L-K formula. Inset: FFT spectra obtained for different temperatures. (b) Oscillatory component of longitudinal electrical conductivity at *T* = 2.5 K calculated with macroscopic Hall resistivity (blue line) or surface channel Hall resistivity (red line).

SdH oscillations are being recognized as a powerful tool in the studies of 3D TIs^9^. Dirac fermions are characterized by a *π* Berry phase (for ordinary metals the Berry phase is equal to zero), and the phase factors of the oscillations reveal the Berry phase values for the system. Observations of SdH oscillations have been reported in some candidate topological materials from the half-Heusler family^19^,^21^,^29^,^32^. For our LuPdBi samples, SdH oscillations were well resolved at temperatures up to 10 K, hence indicating very good quality of the single crystals measured. Figure 7a shows the oscillatory component Δ*ρ*_xx_, obtained by subtracting a background from the *ρ*_xx_(*B*) isotherms. The oscillations are clearly periodic in 1/*B*, however their frequency is very low. Fast Fourier transform (FFT) analysis of these data yielded rather poor quality spectra with broad peaks corresponding to a single temperature-independent frequency ≈10 T, shown in inset to Figure 7a. Obviously, the broadness of FTT peaks comes from low frequency of the oscillations and only less than three periods covered by our measurements. In order to obtain more reliable value of the oscillation frequency *f*_SdH_ we perfomed direct fitting of the standard Lifshitz-Kosevich (L-K) expression where 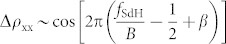 with *β* being a phase shift^9^,^55^. The analysis yielded *f*_SdH_ = 11.9 T and *β* = 0.431(2).

**Figure 8:**
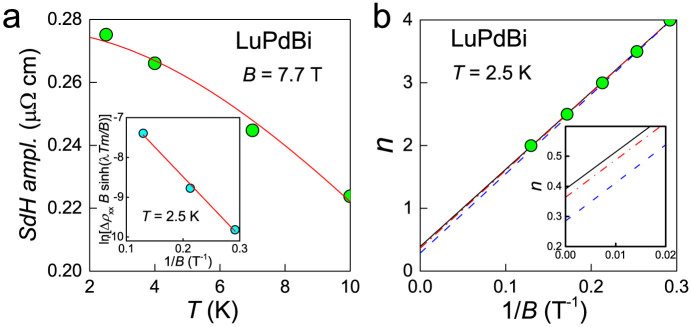
Analysis of the SdH oscillations in LuPdBi. (a) Lifshitz-Kosevich description (solid line) of the temperature dependence of the SdH amplitude. Inset: Dingle plot for the oscillations measured at 2.5 K. Solid straight line determines the Dingle temperature (see the text). (b) Landau-level fan diagram for the SdH oscillations measured at 2.5 K. Fitted straight solid line intersecting the ordinate axis at 0.4 indicates the Berry phase very close to *π*. Dashed (blue) line represents the fit to extrema in conductivity Δ*ρ*_xx_ derived with macroscopic Hall coefficient, *R*_H_. Dot-dashed (red) line corresponds to Δ*σ*_xx_ derived with Hall coefficient of surface channel *R_s_* (see text for explanation). Inset shows the blow up of the area where the fitted lines intercept ordinate axis.

The oscillation frequency is related to the extremal cross section area *A*_F_ of the Fermi surface through the Onsager relation 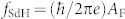, where 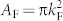. The value of *f*_SdH_ derived for LuPdBi implies the Fermi vector *k*_F_ = 0.019 Å^−1^ that corresponds to a 2D carrier density 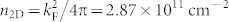 (we assume lifted spin degeneracy). The temperature dependence of the resistivity oscillations amplitude is given by the standard L-K expression Δ*ρ*_xx_(*T*) ~ *λ*(*T*)/sinh(*λ*(*T*)), where 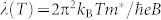 and m* is the effective cyclotron mass^55^. Fitting this equation to the experimental data of LuPdBi (see Figure 8a) one obtains m* = 0.06 *m*_e_, where *m*_e_ is the free electron mass. From the values of *k*_F_ and m*, the Fermi velocity 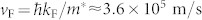 and the Fermi energy 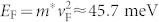 were derived. Since calculation of the Sommerfeld coefficient for a 2D Fermi surface is impossible (it encloses zero volume), we used the formula 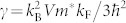 (corresponding to spherical Fermi surface) and obtained the Sommerfeld coefficient equal to 0.003 mJ/mol K^2^. This value is much smaller than 0.75 mJ/mol K^2^ derived from the specific heat data. This finding indicates that the SdH oscillations originate from 2D states Fermi surface or from a tiny 3D Fermi surface, much smaller than the bulk one contributing to the specific heat. If the bulk states were contributing to SdH oscillations one should observe high cyclotron frequency (large *k*_F_) and/or large cyclotron mass, not seen in LuPdBi. Moreover, the Hall concentration of 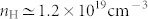 is over 50 times larger than 2.3 × 10^17^ cm^−3^ obtained when we assumed that SdH effect originates from 3D spherical Fermi surface. Such discrepancy in the Hall and SdH concentrations further supports the conjecture that the SdH oscillations in LuPdBi originate from the surface states.

Knowing m*, one can carry out the Dingle analysis shown in the inset to Figure 8a. The lifetime *τ* of the carriers can be found from the Dingle temperature 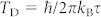. The slope of the linear fit to the data yielded *T*_D_ = 16.9 K, which gave *τ* = 7.2 × 10^−14^ s. Ergo, the mean-free path *l* = *v*_F_*τ* = 26 nm and the surface mobility 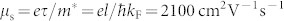 were obtained. Metallicity parameter *k*_F_*l* of LuPdBi is then equal to 5.

Figure 8b presents the Landau-level fan diagram for the SdH oscillations in LuPdBi at 2.5 K. Positions of the extrema in Δ*ρ*_xx_(1/*B*) are marked with circles as a function of their number (*n* for maxima and 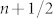 for minima). Though the very small value of *f*_SdH_ allowed to observe only five such extrema, a good linear fit could be performed, with an intercept of 0.391(26), shown as a solid black line. However, the choice of resistance *ρ*_xx_ or conductance *σ*_xx_ for Landau level indexing may be very important and yield different phase factor^9^. Since the Hall resistivity of LuPdBi measured at 2.5 K was *ρ*_xy_(*B* = 9 T) = 0.54 mΩcm, that is very close to *ρ*_xx_ = 0.50 mΩcm, it cannot be ignored in analysis of the SdH oscillations. We thus calculated 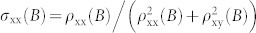 and extracted the oscillatory component Δ*σ*_xx_(1/*B*). These data are shown as blue points in Figure 7b. Straight line fitted to the positions of their extrema (shown as dashed blue line in Figure 8b) yielded the phase factor of 0.285(40) (for clarity the datapoints are not shown).

But the case of LuPdBi is an example (which very likely can be extended to other bulk samples with topologically nontrivial states on their surface) where the oscillatory component of *σ*_xx_(1/*B*) should be derived in a different manner. Our sample, as the temperature dependence of resistivity indicates, contains two parallel conduction channels: metallic and semiconducting (Figure 1b). At low temperatures, the latter makes negligible contribution to the conductivity, but may still play an important role in the Hall effect. However, the SdH oscillations we observe seem to originate from surface carriers. Hence, the Hall resistivity of the surface channel should be used for Δ*σ*_xx_(1/*B*) calculation. It can be easily obtained as *ρ_s_*_,xy_(*B*) = *R_s_B*, where *R_s_* is the Hall coefficient of the surface channel, which can be derived from the formula *R_s_* = *t*/(*en*_2D_), where *n*_2D_ is the surface carrier concentration and *t* is the sample thickness^9^. For our sample of LuPdBi, *R_s_* = 0.0146 mΩcm/T at 2.5 K. After recalculating *σ*_xx_(*B*) as 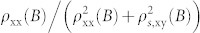, extracting its oscillatory component (shown with red in Figure 7b) and finding positions of its extrema on 1/*B* axis, we could fit another straight line resulting in the phase factor of 0.364(44) (note dot-dashed red line in Figure 8b).

Most remarkably, all the applied methods of determinig the phase factor (we believe that the electronic nature of 3D TIs is most appropriately accounted for in that employing *R_s_*) yielded values very close to 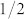, which was theoretically predicted for Dirac fermions. Small departure of the experimental value from 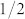 may arise from Zeeman coupling effect^56^,^57^ or small deviation of the energy dispersion of Dirac fermions from the linear one^58^. Nevertheless, the observation in LuPdBi of a *π* Berry phase is another strong indicator of the topologically nontrivial character of the compound.

## Conclusions

We synthesized high-quality single crystals and investigated the electronic properties of a putative 3D topological superconductor LuPdBi. The electrical transport in the compound was found to be driven by two parallel conducting channels: metallic and semiconducting, with the negligible contribution of the latter at low temperatures. The superconductivity emerges at *T*_c_ = 1.9 K and is characterized by the upper critical field *B*_c2_(0) ≤ 2.3 T. It is of type II clean-limit BCS. Remarkably, no feature in the heat capacity is observed near the onset of superconducting state, which rather rules out its bulk character. Instead, we tend to attribute the superconductivity in LuPdBi to the surface states.

The compound shows the linear-in-*B* magnetoresistance, as high as 210% at 2.5 K and over 30% at 300 K (in magnetic field of 9 T). In low magnetic fields, there clearly occurs the weak antilocalization effect. Both features are hallmarks of the topologically protected states. Furthermore, the observed SdH oscillations yield the very small effective mass of 0.06 *m_e_* and the Berry phase very close to *π*, in concert with the topological nature of LuPdBi, characterized by the Fermi surface sheet containing massless, extremely mobile Dirac fermions.

In summary, based on the results of our study, we postulate that at low temperatures, below about 10 K, LuPdBi effectively becomes a topological insulator, with negligible contribution of its bulk to the electronic transport and with the topological nature of its surface states being clearly reflected in the physical properties. The superconductivity in LuPdBi seems to emerge from the topologicaly protected surface states and further confirmation, both experimental and theoretical, of this conjecture is a challenging task for the future.

## Methods

### Material preparation

Single crystals of LuPdBi were grown from Bi flux. The elemental constituents of high purity (Lu: 99.99 wt.%, Pd: 99.999 wt.%, Bi: 99.9999 wt.%), taken in atomic ratio 1:1:12, were placed in an alumina crucible, and sealed under argon atmosphere inside a molybdenum ampule. The reactor was heated slowly up to 1300°C and kept at this temperature for 15 hours. Next, it was cooled down to 1000°C at a rate of 3°C/hour, and then to room temperature at a rate of 5°C/hour. The crucible with the charge was extracted from the molybdenum ampule, covered with another alumina crucible filled with silica wool and resealed in an evacuated quartz tube. Then, the tube was heated up to 900 K at which point the excess of Bi flux was removed by centrifugation. The crystals obtained by this technique had a shape of cubes or plates, with dimensions up to 0.5 × 0.5 × 0.5 mm^3^ or 0.1 × 0.5 × 3 mm^3^, respectively. They had metallic luster and were stable against air and moisture.

### Material characterization

Powdered single crystals of LuPdBi were characterized at room temperature by powder X-ray diffraction (PXRD) carried out using an X'pert Pro PANanalitical diffractometer with Cu-K*α* radiation. The crystal structure refinements and the theoretical PXRD pattern calculations were done employing the FULLPROF program^59^. Quality of the samples studied was also verified by single crystal X-ray diffraction (SCXRD) performed at room temperature on an Oxford Diffraction Xcalibur four-circle diffractometer equipped with a CCD camera and using Mo-K*α* radiation. The SCXRD data analysis was done using the program package SHELXL-97^60^. The results of PXRD and SCXRD investigations are presented in Supplementary Material.

Chemical composition of the LuPdBi crystals was examined on a FEI scanning electron microscope (SEM) equipped with an EDAX Genesis XM4 spectrometer. The specimens were glued to a SEM stub using carbon tape. The crystals were found homogeneous and free of foreign phases, with the chemical compositions very close to the ideal one (for details see Supplementary Material).

### Physical measurements

Electrical resistivity and Hall measurements were carried out from 0.4 K to 300 K in applied magnetic fields up to 9 T using a conventional ac four-point technique implemented in a Quantum Design PPMS platform. Dimensions of samples were: 0.010 × 0.051 × 0.227 cm^3^, for *ρ_xx_*, and 0.0067 × 0.051 × 0.1524 cm^3^ for *ρ_xy_* measurements. Current and voltage leads were 50 *μm* thick silver wires attached to the parallelepiped-shaped specimens with silver paste and additionally spot welded. The heat capacity was measured on 10.6 mg collection of single crystals, in the temperature interval 0.4–5 K by relaxation time method using also the PPMS platform. DC magnetization and AC magnetic susceptibility measurements were performed on 30.1 mg collection of single crystals, in the temperature range 1.62–2.6 K in weak magnetic fields up to 2 mT employing a Quantum Design MPMS-XL SQUID magnetometer.

## Author Contributions

D.K. and P.W. designed the research project, O.P. prepared the samples, O.P. and P.W. performed the physical measurements and analyzed the data. All the authors discussed the experimental results and wrote the manuscript.

## Supplementary Material

Supplementary Information Supplementary Material
