# Supplementary material for: Shubnikov - de Haas oscillations, weak antilocalization effect and large linear magnetoresistance in the putative topological superconductor LuPdBi
Source: Sci Rep. 2015 Mar 17;5:9158. doi: 10.1038/srep09158 (PMC4361847; doi:10.1038/srep09158)
Supplement: Supplementary Information — Supplementary Material [file srep09158-s1.pdf]

SUPPLEMENTARY MATERIAL

Shubnikov-de Haas oscillations, weak antilocalization effect and large linear magnetoresistance in the putative topological superconductor LuPdBi

Orest PAVLOSIUK, Dariusz KACZOROWSKI and Piotr WIŚNIEWSKI*

* Correspondence to: p.wisniewski@int.pan.wroc.pl

MATERIAL CHARACTERIZATION

X-ray diffraction

The PXRD results (see Fig. S1) confirmed a single-phase character of the obtained single crystals of LuPdBi. The X-ray diffraction pattern can be fully indexed within the $F\bar{4}3m$ space group, characteristic of half-Heusler compounds, and yields the cubic lattice parameter $a = 6.565(1)$ Å. This value is in perfect accord with the literature value 6.566(1) Å determined for polycrystalline sample,[1] and the value 6.56 Å reported for thin films of LuPdBi.[2] It is however markedly different from the lattice parameter of 6.63 Å stated by Xu et al. for their powdered single crystals.[3] As can be inferred from Fig. S1, the experimental PXRD pattern of our single-crystalline LuPdBi can be very well modeled with the MgAgAs-type crystal structure with the Lu atoms located at the crystallographic $4a$ $(0, 0, 0)$ sites, the Pd atoms occupying the $4c$ $(1/4, 1/4, 1/4)$ sites, and the Bi atoms placed at the $4b$ $(1/2, 1/2, 1/2)$ sites. The same structural model was recently shown to account well for the PXRD data of powdered single crystals of YPdBi and YPtBi.[4]

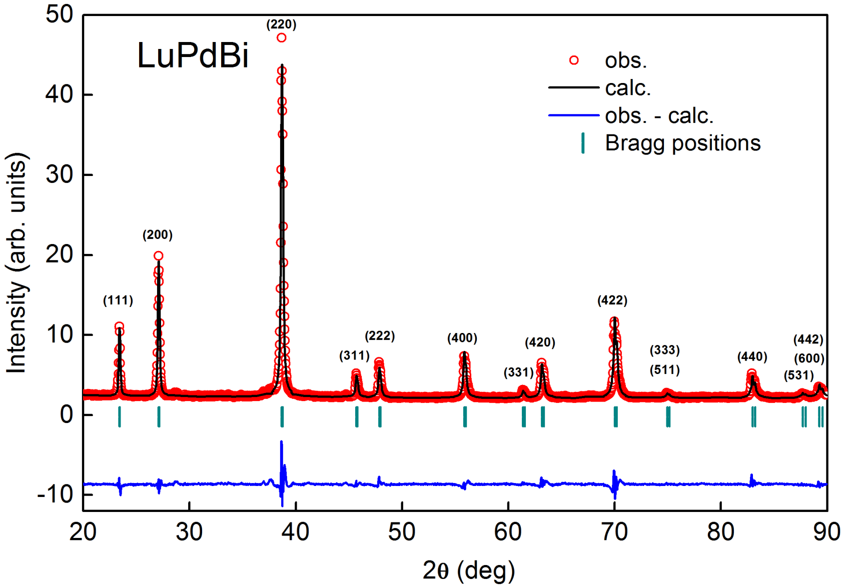

Figure S1. XRD pattern for powdered single crystals of LuPdBi.

It is worth noting that despite the aforementioned divergence in the values of the lattice parameter a, the X-ray diffractogram of LuPdBi presented in Ref. 3 appears very similar to our result displayed in Fig. S1. Small differences in the relative intensities of a few Bragg peaks [e.g., (311), (222), (420)] might be attributed to a certain level of atomic disorder in the sample studied by Xu at al., since our PXRD data were refined to a small value of the residual Bragg factor $R = 4.61\%$, with full occupancies of all the crystallographic positions and no structural disorder (see also the discussion presented in Ref. 4).

The crystal structure of the studied samples of LuPdBi was also checked by the single crystal X-ray diffraction method. Details on the performed refinement are gathered in Table S1. The positional data and the equivalent isotropic displacement parameters are given in Table S2. The so-refined lattice parameter $a = 6.5683(10)$ Åis very close to that derived from the PXRD data, and thus significantly smaller than the result reported for single-crystalline LuPdBi by Xu at al.[3]

Table S1. Crystal data and structure refinement for LuPdBi.

Formula weight	490.35
Temperature	295(2) K
Wavelength	0.71073 Å
Crystal system	Cubic
Space group	F $\bar{4}$3m
Unit cell parameter	a = 6.5683(10) Å
Volume	283.37(13) $Å^3$
Z	4
Density (calculated)	11.494 Mg/m^3
Absorption coefficient	102.423 mm^{-1}
F(000)	800
Crystal size	0.04 x 0.03 x 0.03 mm^3
Theta range for data collection	5.377 to 26.497°.
Index ranges	-8<=*h*<=8, -8<=*k*<=8, -8<=*l*<=8
Reflections collected	627
Independent reflections	48 [R(int) = 0.0808]
Completeness to theta = 25.242°	96.0 %
Absorption correction	Numerical
Max. and min. transmission	0.137 and 0.060
Refinement method	Full-matrix least-squares on F^2
Data / restraints / parameters	48 / 0 / 5
Goodness-of-fit on F^2	1.047
Final R indices [I>2sigma(I)]	R_1 = 1.76%, wR_2 = 4.53%
R indices (all data)	R_1 = 1.76%, wR_2 = 4.53%
Absolute structure parameter	-0.043(16)
Extinction coefficient	0.0048(7)
Largest diff. peak and hole	1.079 and -1.524 e/$Å^3$

Table S2. Atomic coordinates and equivalent isotropic displacement parameters $U(eq)$ for LuPdBi. $U(eq)$ is defined as one third of the trace of the orthogonalized U^{ij} tensor.

**Atom**	*x*	*y*	*z*	*U(eq)* [$Å^2 \times 10^3$]
**Lu**	**0**	**0**	**0**	**9(2)**
**Pd**	**0.25**	**0.25**	**0.25**	**10(2)**
**Bi**	**0.5**	**0.5**	**0.5**	**7(1)**

Energy-dispersive X-ray spectroscopy

Examples of the EDX spectrum and the SEM image obtained for the single crystals of LuPdBi is presented in Fig. S2. The chemical composition derived from these data is $Lu_{32.00(32)}Pd_{33.93(33)}Bi_{34.07(34)}$, in a very good accord with the ideal equiatomic one.

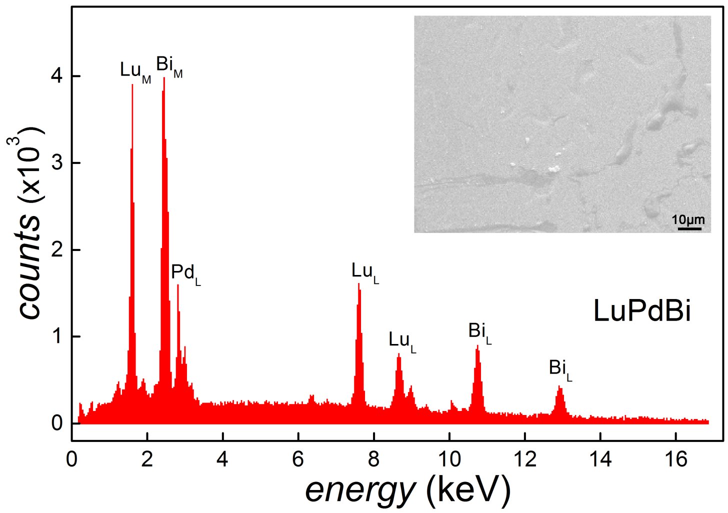

Figure S2. SEM-EDX results for single-crystalline LuPdBi.

ELECTRONIC AND THERMODYNAMIC PROPERTIES

WAL parameters

The temperature dependencies of the weak antilocalization parameters $\frac{\eta e^2}{2\pi^2\hbar}$ and L_φ (together with their uncertainties $d(\frac{\eta e^2}{2\pi^2\hbar})$ and dL_φ) obtained from fitting the Hikami-Larkin-Nagaoka (HLN) formula (Eq. 1 in the main text) to the experimental $\sigma(B)$ data of LuPdBi are collected in Table S3.
When both parameters were free to vary in fitting procedure, the values given in second and fourth columns of the Table S3 were obtained. However the L_φ varied with temperature in irregular way and both parameters were strongly dependent on each other, as shown by statistical dependency values in fifth column. Thus we could not consider all these values of $\frac{\eta e^2}{2\pi^2\hbar}$ and L_φ as reliable. Reasoning that the WAL model is most adequate at lowest temperature, where the contribution from the bulk semiconducting channel is the smallest, we repeated the fitting with the value of $\frac{\eta e^2}{2\pi^2\hbar}$ fixed at the value derived at $T = 2.5\,\mathrm{K}$. Resulting values of L_φ are collected in sixth column of the Table. It is worth noting that their errors are now smaller by about one order of magnitude. These values decrease monotonously with increasing temperature, which seems to reflect the increasing of the bulk channel contribution.

TABLE S3. WAL parameters for sheet conductance of LuPdBi at different temperatures.

T(K)	$\frac{\eta e^2}{2\pi^2\hbar}$	$d(\frac{\eta e^2}{2\pi^2\hbar})$	L_φ	dL_φ	dependency $(\frac{\eta e^2}{2\pi^2\hbar}, L\varphi)$	$L_\varphi\|_{\eta(T=2.5K)}$	$dL_\varphi\|_{\eta(T=2.5K)}$
2.5	-153.7	21.8	49.6	2.2	0.9976	49.6	0.1
4	-340.5	149.1	33.1	4.0	0.9996	41.3	0.1
7	-99.8	21.6	43.5	2.8	0.9986	38.4	0.1
10	-65.6	12.9	48.8	3.0	0.9977	37.9	0.1
50	-49.6	7.4	44.7	2.0	0.9984	32.6	0.1
150	-8.8	0.5	52.1	0.9	0.9971	23.6	0.1

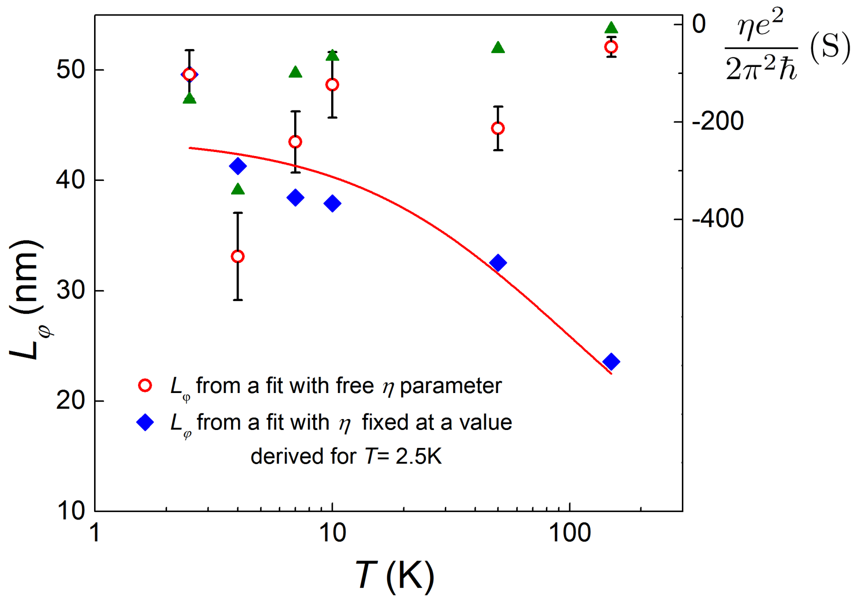

Figure S3. Temperature variations of the WAL parameters for single-crystalline LuPdBi. Green triangles represent values of $\frac{\eta e^2}{2\pi^2\hbar}$, circles and diamonds correspond to L_φ obtained from two fitting procedures described in text.

For more clarity we plotted the values of $\frac{\eta e^2}{2\pi^2\hbar}$ and L_φ resulting from both fitting procedures, versus temperature, as shown in Fig. S3.

The temperature variation of the phase coherence length $L\varphi|_{\eta(T=2.5K)}$ (resulting from the HLN fit with $\frac{\eta e^2}{2\pi^2\hbar}$ fixed at the value derived at $T = 2.5\,\mathrm{K}$.) can be roughly described by the formula given in Ref. 3 (represented in Fig. S3 by the solid red curve), however such a fit yields the electron-phonon interaction parameter close to zero.

The $\frac{\eta e^2}{2\pi^2\hbar}$ parameter derived in Ref. 3 was about an order of magnitude smaller than that obtained on our sample, whereas corresponding values of L_φ were about twice as large as our $L\varphi|_{\eta(T=2.5K)}$ data. This is rather a good agreement, taking into account different electronic character of the samples examined in our work and that described in Ref. 3. It seems to be a common feature of half-Heusler compounds that the values of parameter $-\eta$ are of order $10^5 - 10^6$, thus much larger than 1/2 expected for 2D TI systems. Apart from LuPdBi it has very recently been observed in samples of LuPtSb,[5] YPtBi and LuPtBi.[6] Authors of all these papers attributed this fairly unexpected result to contributions from bulk or side wall conductivity channels.

Hall parameters

In concert with the previous report,[3] the Hall carrier concentration in the single crystal investigated in our work was found temperature independent below 50 K and then to increase with increasing temperatures. However, in the entire temperature range covered, the magnitude of n_H determined in our study is approximately four times smaller than that estimated by Xu et al. (their result was $n_\mathrm{H} \simeq 4.8 \times 10^{19}\mathrm{cm}^{-3}$ at low temperatures). Remarkably, our findings are completely different as regards the Hall carrier mobility. For our sample, μ_H was found to gradually decrease with increasing temperature from the value $2404\,\mathrm{cm}^2\mathrm{V}^{-1}\mathrm{s}^{-1}$ at $T = 2.5\,\mathrm{K}$ to $573\,\mathrm{cm}^2\mathrm{V}^{-1}\mathrm{s}^{-1}$ at 300 K, while Xu et al. reported an increase from 330 to $380\,\mathrm{cm}^2\mathrm{V}^{-1}\mathrm{s}^{-1}$ in the same temperature interval.

Heat capacity

The striking result reported in Ref. 3 is the occurrence of a pronounced λ-shaped anomaly in the specific heat of LuPdBi that coincides with the superconducting transition. Consequently, Xu et al. ascribed the superconductivity in their single crystals to the bulk. This result markedly differs from the behavior of our single crystals, where no visible feature in $C(T)$ was found near T_c, despite the formation of superconducting state was convincingly proved by means of the magnetic and electrical transport measurements. This finding led us to the conclusion that the

superconductivity in our samples of LuPdBi is confined to the surface, with the superconducting condensate volume negligibly small as compared to the bulk. The result obtained by Xu et al. differs also from the properties of closely related systems LuPtBi and YPtBi,[7,8] for which the specific heat was found featureless at the superconducting transition. For the latter compound we recently confirmed the lack of any anomaly at T_c in our own investigation carried out on high-quality single crystals.[9]

The observed discrepancy might be related to the different electronic character of the single crystals studied in our work and those investigated in Ref. 3. Comparison of the temperature-dependent electrical resistivity suggests that our samples are much more semimetallic than the predominantly semiconducting one reported by Xu et al. In this context, however, the observed over-an-order-of-magnitude discrepancy between the Sommerfeld coefficient $\gamma = 0.75 \mathrm{mJ/mol\,K^2}$ derived in the present work and $\gamma = 11.9 \mathrm{mJ/mol\,K^2}$ found by Xu et al. becomes most surprising. Here, again, one should note that the electronic specific heat of the related semimetallic nonmagnetic bismuthides, like LuPtBi and YPtBi (Refs. 7–10), and antimonides, like LuPtSb, YPdSb and YPtSb (Ref. 11) is similar to that established by us for LuPdBi.

References

[1] Haase, M. G., Schmidt, T., Richter, C. G., Block, H. & Jeitschko, W. Equiatomic Rare Earth (Ln) Transition Metal Antimonides LnTSb (T=Rh, lr) and Bismuthides LnTBi (T=Rh, Ni, Pd, Pt). *J. Solid State Chem.* **168**, 18–27; *DOI:10.1006/jssc.2002.9670* (2002).

[2] Shan, R. *et al.* Electronic and crystalline structures of zero band-gap LuPdBi thin films grown epitaxially on MgO(100). *Appl. Phys. Lett.* **102**, 172401; *DOI:10.1063/1.4802795* (2013).

[3] Xu, G. *et al.* Weak Antilocalization Effect and Noncentrosymmetric Superconductivity in a Topologically Nontrivial Semimetal LuPdBi. *Sci. Rep.* **4**, 5709; *DOI:10.1038/srep05709* (2014).

[4] Nowak, B. & Kaczorowski, D. NMR as a Probe of Band Inversion in Topologically Nontrivial Half-Heusler Compounds. *J. Phys. Chem. C* **118**, 18021; *DOI:10.1021/jp505320w* (2014).

[5] Hou, Z. *et al.* Transition from semiconducting to metallic-like conducting and weak antilocalization effect in single crystals of LuPtSb., preprint arXiv:1501.06714 (2015). URL `http://arxiv.org/abs/1501.06714` Date of access: 15/02/2015.

[6] Shekhar, C., Kampert, E., Förster, T., Nayak, A.K., Nicklas, M. & Felser, C. Large linear magnetoresistance and weak anti-localizatio n in Y(Lu)PtBi topological insulators. preprint arXiv:1502.00604 (2015). URL `http://arxiv.org/abs/1502.00604` Date of access: 15/02/2015.

[7] Mun, E. *et al.* Magnetic-field-tuned quantum criticality of the heavy-fermion system YbPtBi. *Phys. Rev. B* **87**, 075120; *DOI:10.1103/PhysRevB.87.075120* (2013).

[8] Pagliuso, P. *et al.* Low temperature specific heat of YBiPt. *APS March Meeting 2013, Baltimore*, Abstract ID: BAPS.2013.MAR.B13.13 (2013). URL `http://meeting.aps.org/Meeting/MAR13/Session/B13.13` Date of access:15/01/2015.

[9] Pavlosiuk, O., Wiśniewski, P. & Kaczorowski, D. Superconductivity and Shubnikov-de Haas oscillations in the noncentrosymetric half-Heusler compound YPtBi. SCES'14 Conference, Grenoble Abstract Mo-101 (2014). URL `https://www.ill.eu/nc/fr/presse-et-infos/events/sces-2014/abstract-booklet/?cid=45039&did=69737&sechash=f08eb1b1` Date of access:15/01/2015.

[10] Riedemann, T. M. *Heat capacities, magnetic properties, and resistivities of ternary RPdBi alloys where R = La, Nd, Gd, Dy, Er, and Lu.* MSc thesis, Iowa State University, Ames *DOI:10.2172/251374* (1996).

[11] Nowak, B. & Kaczorowski, D. Nonmetallic behaviour in half-Heusler phases YPdSb, YPtSb and LuPtSb. *Intermetallics* **40**, 28–35; *DOI:10.1016/j.intermet.2013.04.001* (2013).
